# Detection of potential safety signals related to the use of remdesivir and tocilizumab in the COVID era during pregnancy, resorting to open data from the FDA adverse event reporting system (FAERS)

**DOI:** 10.3389/fphar.2024.1349543

**Published:** 2024-01-31

**Authors:** Beatriz Marinho Silva Romão, Felipe Vieira Duval, Elisângela Costa Lima, Fabrício Alves Barbosa da Silva, Guacira Correa de Matos

**Affiliations:** ^1^ Observatory of Medicines Surveillance and Use, Pharmacy School, Federal University of Rio de Janeiro, Rio de Janeiro, Brazil; ^2^ Electric Power Research Center, Rio de Janeiro, Brazil; ^3^ Scientific Computing Program, Oswaldo Cruz Foundation, Rio de Janeiro, Brazil

**Keywords:** pharmacovigilance, data mining, adverse drug events, FAERS, COVID-19, pregnancy complications

## Abstract

**Background:** The in-hospital treatment for COVID-19 may include medicines from various therapeutic classes, such as antiviral remdesivir and immunosuppressant tocilizumab. Safety data for these medicines are based on controlled clinical trials and case reports, limiting the knowledge about less frequent, rare or unique population adverse events excluded from clinical trials.

**Objective:** This study aims at analyzing the reports of Adverse Drug Events (ADEs) related to these two medicines, focusing on events in pregnant women and foetuses.

**Methods:** Data from the open-access FDA Adverse Event Reporting System (FAERS) from 2020 to 2022 were used to create a dashboard on the Grafana platform to ease querying and analyzing report events. Potential safety signals were generated using the ROR disproportionality measure.

**Results:** Remdesivir was notified as the primary suspect in 7,147 reports and tocilizumab in 19,602. Three hundred and three potential safety signals were identified for remdesivir, of which six were related to pregnant women and foetuses (including abortion and foetal deaths). Tocilizumab accumulated 578 potential safety signals, and three of them were associated with this population (including neonatal death).

**Discussion:** None of the possible signals generated for this population were found in the product labels. According to the NIH and the WHO protocols, both medicines are recommended for pregnant women hospitalized with COVID-19.

**Conclusion:** Despite the known limitations of working with open data from spontaneous reporting systems (e.g., absence of certain clinical data, underreporting, a tendency to report severe events and recent medicines) and disproportionality analysis, the findings suggest concerning associations that need to be confirmed or rejected in subsequent clinical studies.

## 1 Introduction

COVID-19 is a viral disease that became a pandemic in March 2020. Mass immunization was able to reduce hospital admissions due to the disease, with most of the critical cases recorded among unvaccinated patients hospitalized due to the disease (Uzun et al., 2022).

The in-hospital treatment protocols for COVID-19 from the United States National Institutes of Health (2023) and the World Health Organization (2023) are not unanimous in their recommendations and include using anticoagulants, corticosteroids, antivirals, IL-6 inhibitors or JAK inhibitors. The medicines choices and combinations vary between the protocols and according to the severity of each patient’s clinical condition.

In the literature, tocilizumab (an IL-6 inhibitor approved by the European Medicines Agency for the treatment of rheumatoid arthritis in 2009 and by the US Food and Drug Administration [US FDA] for COVID-19 treatment in 2020) and remdesivir (an antiviral approved by the US FDA in 2020) are two of the medicines included in the guidelines and are associated with few serious Adverse Drug Events (ADEs) during treatment for COVID-19 (Fan et al., 2020; Tleyjeh et al., 2021; Angamo et al., 2022; Yu et al., 2022). However, as data from these studies are based on controlled clinical trials, observational studies and case reports, less frequent events may not have been detected.

In cases of treatment in pregnant or lactating women, both protocols indicate the same medications as for non-pregnant individuals, but with some reservations. The WHO therapeutic guide states that tocilizumab may cross the placental barrier, but that foetal harm is uncertain. Remdesivir is indicated for this population in both protocols, providing that it is a decision between the patient and the health team. There is lack of available data on ADEs in pregnant individuals (NIH, 2023; World Health Organization, 2023).

Serious, unknown or rare ADEs aggregated in medicines regulatory agencies’ pharmacovigilance databases can generate potential safety signals through data mining (Beninger, 2018). As an example, we highlight the potential hepatotoxic (Montastruc, Thuriot, Durrieu, 2020) and nephrotoxic (Gérard et al., 2021) effects of remdesivir, which have proven to be significant associations through pharmacovigilance studies in large databases. Such findings are particularly important when it comes to events related to populations often excluded from clinical trials, such as pregnant women. Despite the absence of prior safety data, the need for therapy leads to discovering adverse events related to pregnancy and the fetus after marketing (Cavadino et al., 2021; Trifirò et al., 2021).

A safety signal is a data set that suggests the existence of a correlation between the medication and the ADE, although not necessarily a causal relationship. It is an association hypothesis that justifies further epidemiological studies to confirm the hypotheses raised by these analyses. By itself, it does not confirm the existence of any risk; thus, other data and evidence are necessary to support or refute the association (Montastruc et al., 2011; Bihan et al., 2020).

This study aimed at analyzing the reports of all type of ADEs related to remdesivir and tocilizumab notified to the FDA Adverse Event Reporting System (FAERS) pharmacovigilance database, focusing on events related to pregnancy and the foetus, by generating potential safety signals.

## 2 Materials and methods

This quantitative and descriptive study analyzes anonymized secondary data from Individual Case Safety Reports (ICSRs) notified to FAERS.

The files in ASCII format corresponding to all quarters of 2020, 2021 and 2022 were downloaded. In addition to the report data, these files contain explanatory material indicating the columns for creating a relational database. With this description, tables were created using a relational database management system: PostgreSQL.

The search in the database for the medications under analysis was performed using both the International Nonproprietary Name (INN) and the Veklury^®^ (remdesivir) and Actemra^®^ (tocilizumab) brand names. In order to avoid repetitions of reports with multiple updates, only the most recent version of the notification was considered in this study. Reports extracted during 2020, 2021 or 2022 but with an event date outside the range were excluded.

To insert the data into the database, a program was written in the Ruby language to transfer the data from the text (TXT) files into their corresponding SQL table. We created one table for each entity in the FAERS ASCII Entity Relationship Diagram (ERD).

Using the Grafana platform free interface, a dashboard containing tables and graphs was created with data from the databases. Links with snapshots from the dashboards and program scripts are available as Supplementary Material.

The dashboard allowed seeing all the ADEs aggregated in the database during the period analyzed, the cumulative frequency of each ADE, and the frequency of ADEs reported for the medications under analysis. In accordance with the aforementioned explanatory material, the data provided by FAERS are harmonized by the agency itself according to the “Preferred Term”-level medical terminology describing the event, using the Medical Dictionary for Regulatory Activities, 2022 (MedDRA) in all reports. No “Preferred Term” was excluded. Data on the role of the medicines in the event, the type of notifier, the outcome of the reports, and the indication for using the medicines were also analyzed.

To investigate the existence of possible safety signals, the approach chosen was Reporting Odds Ratio (ROR), calculated using the following formula:

$$ROR=\frac{A/B}{C/D}$$

where A is the number of reports involving medicine P and event R; B is the number of reports involving medicine P and all other events except R; C is the number of reports involving event R and all medicines except P; and D is the number of reports involving all events except R, related to all medicines except P (European Medicines Agency, 2016). The choice is justified by its simplicity as well as being one of the standard analyses recognized by health authorities for signal detection.

ROR calculations were performed for every medicine-event pair with a frequency ≥3, with differentiation for those with frequencies ≥5. If the event was unrelated to any other medicine, it was impossible to calculate ROR (C = 0 in the contingency table), and an arbitrary value of 99.9 was assigned to indicate the presence of a possible safety signal. Any event with frequency ≥3 whose ROR lower confidence interval was >1 was defined as a signal (European Medicines Agency, 2016).

Medicine-event pairs were only formed for events where the medicines under study were classified as the primary suspects in the reports to increase specificity. In the ROR calculation, all medicines (in all pharmaceutical forms, concentrations and commercial or active ingredient names) and all events reported to FAERS from 2020 to 2022 were considered in the sample universe, totaling 272,247 medicines and 18,580 different adverse events.

The tables created in the dashboard were downloaded in.csv format and processed in a Microsoft Excel^®^ spreadsheet editor to calculate the 95% Confidence Interval (95%CI) to determine which events were considered potential safety signals.

An additional analysis was conducted to gather more information (use indication, age at the time of the event, gender and concurrent medicine use) in the reports that contained the ADEs considered possible safety signals for tocilizumab and remdesivir to describe those associated to pregnancy.

The signals identified were categorized and aggregated according to the MedDRA System Organ Class (SOC) classification and were also compared to the ADEs reported on the product labels on the FDA website (Drugs FDA, 2023).

## 3 Results

**FIGURE 1:**
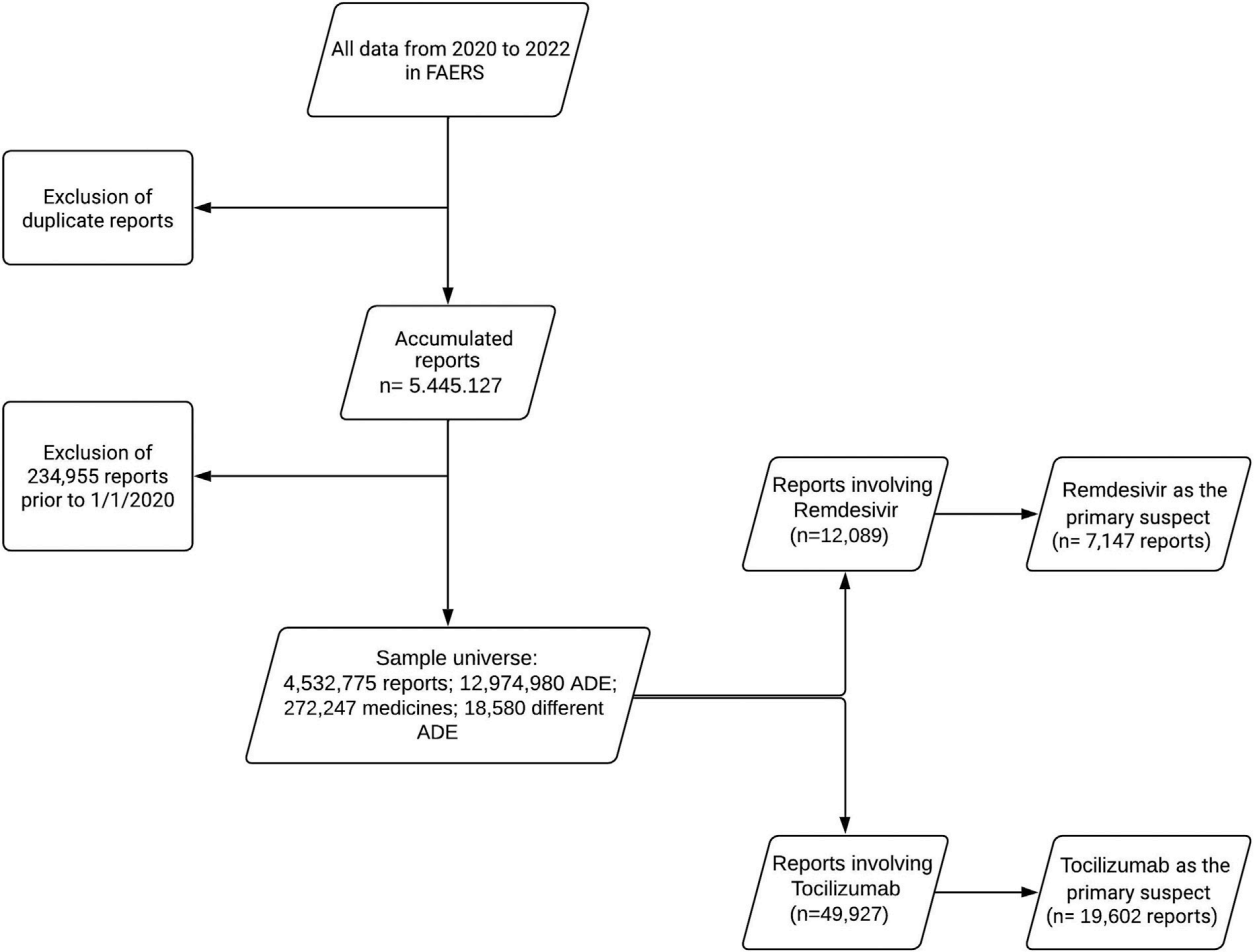
Flowchart of the FAERS database debugging process (2020–2022).

In addition to the duplicates, we excluded another 234,955 reports with dates prior to 01/01/2020 in the database. The analysis included 4,532,775 reports during the period analyzed in the entire database. There were 12,974,980 ADEs among these reports. Figure 1 schematically summarizes the quarterly FAERS database debugging process and the number of notifications and events included in the study.

Out of the 12,089 reports involving remdesivir, in 59.1% (*n* = 7,147) of the cases the medicine was classified as the primary suspect for the ADEs reported. Only considering these latter reports, pharmacists were the leading notifiers, accounting for 4,569 instances (63.9%). Although the COVID-19 treatment is the only approved indication for the medicine, 8.2% of the report indications were for other diseases and clinical conditions. In the same report, the primary suspected medicine can have more than one or no use indication.

In the reports involving tocilizumab, only 39.3% (*n* = 19,602) out of 49,927 reports had the medicine classified as the primary suspect for the events. The consumers’ role stands out, as they notified 8,112 (41.4%) out of the 19,602 reports. Table 1 provides additional information about the notifiers for both medicines. Regarding use indications, only 12.5% of the reports where tocilizumab was the primary suspect were related to its use for treating COVID-19, whereas 45.5% were for autoimmune diseases.

**TABLE 1:** Reports of ADEs related to remdesivir and tocilizumab by notifier categories. FAERS (2020–2022).

Notifier	Medications			
	Remdesivir		Tocilizumab	
	n	%	n	%
Physician	1,272	17.8	4,169	21.3
Pharmacist	4,569	63.9	1,437	7.3
Other health professional	679	9.5	5,433	27.7
Lawyer	2	0	2	0
Consumer	401	5.6	8,112	41.4
Missing	224	3.1	449	2.3
**Totals**	**7,147**	**100**	**19,602**	**100**

Congenital anomalies were reported as an outcome in 10 reports for remdesivir and in 19 reports for tocilizumab. Death was related to 1,840 reports for the antiviral and to 1,740 for the monoclonal antibody. Other outcomes are detailed in Table 2. A report can have one, none or more associated outcomes.

**TABLE 2:** Outcomes of the notifications in which remdesivir and tocilizumab were the primary suspects, by ADEs. FAERS (2020–2022).

Outcomes	Medications			
	Remdesivir		Tocilizumab	
	n	%	n	%
Death	1,840	24.7	1,740	12.0
Congenital Anomaly	10	0.1	19	0.1
Life-Threatening	420	5.6	429	3.0
Disability	95	1.3	459	3.2
Required Intervention to Prevent Permanent Impairment/Harm	124	1.7	21	0.1
Hospitalization - Initial or Prolonged	1,203	16.1	3,545	24.5
Other Serious Ones (Important Medical Events)	3,761	50.5	8,241	57.0
**Totals**	**7,453**	**100**	**14,454**	**100**

Comparing both medicines analyzed, tocilizumab accumulated more reports (as the primary suspect) and ADEs (19,602; 102,289, respectively) than remdesivir (7,147; 16,184, respectively). These and other data are available in the snapshots in the Supplementary Material section.

### 3.1 Detection of signals for remdesivir

For remdesivir, 1,484 different types of ADEs were found, of which 303 were classified as possible safety signals. Among these, 231 had more than five reports related to the medicine-event pair (“a” ≥5).

Four (4) signals were grouped in the category of clinical conditions in pregnancy, childbirth and perinatal period, and two (2) signals from other categories were related to medication use safety during pregnancy (Table 3).

**TABLE 3:** Potential safety signals related to pregnancies, foetuses and neonates found for remdesivir. FAERS (2020–2022).

SOC^a^	Signal	Frequency of drug-event pair	ROR^b^ (95%CI)
Pregnancy, Puerperium and Perinatal Conditions	Abortion	3	5.2 (1.7–16.1)
	Foetal death	7	6.3 (3.0–13.3)
	Premature delivery	11	2.0 (1.1–3.6)
	Premature labor	4	4.2 (1.6–11.1)
Injury, Poisoning and Procedural Complications	Maternal drugs affecting foetus	3	10.8 (3.4–33.7)
	Maternal exposure during pregnancy	70	2.3 (1.8–2.9)

^a^ System Organ Class.

^b^ Reporting Odds Ratio.

**FIGURE 2:**
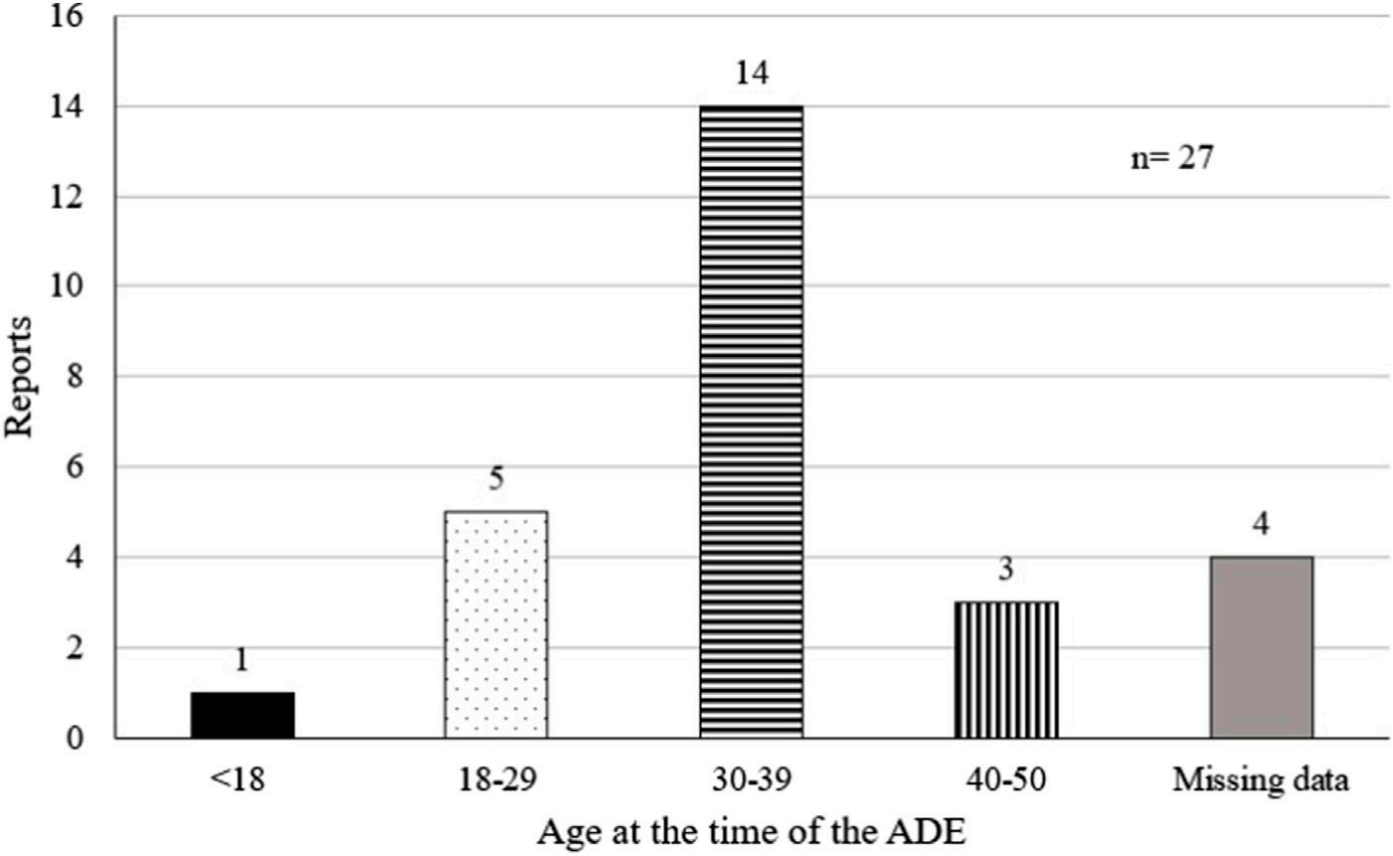
Age of the pregnant women in the reports involving remdesivir and the abortion, foetal death, premature delivery and premature labor events.

We identified 27 reports when analyzing the reports from which the “abortion,” “foetal death,” “premature delivery” and “premature labor” possible signals related to remdesivir were derived. Three (3) reports did not have data related to the person’s age at the time of the event (11.1% missing data). Figure 2 illustrates the distribution of these reports in relation to age, which ranged from 16 to 42 years old.

In addition to these ADEs, all of them contained at least one event indicating use of the medicine during pregnancy, such as “maternal exposure during pregnancy,” “foetal exposure during pregnancy,” “maternal condition affecting the foetus” or “exposure during pregnancy.”

In all 27 reports analyzed, remdesivir was indicated for treating COVID-19. The use of other medicines was associated with the reports in 18 out of the 27 cases. All of this data for both medicines are available in snapshots in the Supplementary Material section.

### 3.2 Detection of signals for tocilizumab

A total of 3,552 adverse events related to tocilizumab were identified, of which 578 were classified as potential safety signals, with 491 having “a” ≥5.

The list of all events found for both medicines and the ROR values, as well as “a,” “b,” “c” and “d,” are also available in the Grafana dashboard snapshots in the Supplementary Material section.

Although no signals were classified in the category of clinical conditions in pregnancy, childbirth and perinatal period, three signals related to gestation, neonates and foetuses were found (Table 4).

**TABLE 4:** Potential safety signals related to pregnancies, foetuses and neonates found for tocilizumab. FAERS (2020–2022).

SOC^a^	Signal	Frequency of drug-event pair	ROR^b^ (95%CI)
General disorders and administration site conditions	Death neonatal	8	4.1 (2.0–8.2)
Injury, Poisoning and Procedural Complications	Maternal exposure timing unspecified	26	6.7 (4.5–9.9)
	Maternal exposure during pregnancy	506	2.6 (2.4–2.9)

^a^ System Organ Class.

^b^ Reporting Odds Ratio.

When analyzing the reports that led to the “death neonatal” potential signal, we identified 8 reports. They all had the “age” field blank (100% missing data). Only two reports had gender data (female), resulting in 75% missing data in this category.

The tocilizumab use indications in these reports varied between “product used for unknown indication” (*n* = 7) and “rheumatoid arthritis” (*n* = 1).

All 8 reports had another associated “a” event indicating maternal exposure to the medicine during pregnancy: “foetal exposure during pregnancy.” Regarding the report of other concomitant drugs, all of them had more than one associated medication.

## 4 Discussion

### 4.1 Potential signals for tocilizumab

Two out of the three possible signals identified for tocilizumab (“unspecified duration of maternal exposure” and “maternal exposure during pregnancy”) are events that indicate use of the medicine during pregnancy rather than adverse reactions to the medicine.

The frequency with which the “tocilizumab-maternal exposure during pregnancy” and “tocilizumab-unspecified duration of maternal exposure” pairs appear is much higher (n = 506 and *n* = 26, respectively) than the association between the medicine and “death neonatal” (*n* = 8). Nonetheless, further analyses are important to investigate whether there are associations between tocilizumab and other types of foetal harm that were not observed in this study.

The product’s label, revised in December 2022, warns about the potential risk of foetal harm. Although there is not enough available data to determine this risk, it is known that monoclonal antibodies are transported across the placenta, especially during the third trimester of pregnancy, and that they can affect the uterine immune response. According to the manufacturer, data from animal studies show that this medicine can cause miscarriages and neonatal deaths. The document also states that there is no record of the medicine’s presence in breast milk, although immunoglobulins are in fact found (Actemra, 2023).

In the NIH COVID-19 treatment guideline (2023), tocilizumab is recommended for pregnant women, with the caveat that the decision should be made considering the risks and benefits. Breastfeeding is also indicated as a safe practice.

None of the reports in which the “death neonatal” event occurred were explicitly related to using the medication for the treatment of COVID-19, whereas the use indication was rheumatoid arthritis in 33.3% of them. Furthermore, more than one-third of the tocilizumab use indications (as primary suspect) in the database were for treating rheumatological diseases.

Therefore, it is possible that this event is related to the disease itself and not to the medicine, as rheumatoid arthritis and other autoimmune diseases increase the risk of preterm delivery (Kolstad et al., 2020; Falcon et al., 2023) and perinatal morbidity and mortality (Borchers et al., 2010).

### 4.2 Potential signals for remdesivir

Approved for human use in the United States in 2020, remdesivir had the highest number of signals related to the foetus and pregnancy in comparison with tocilizumab. However, the product’s label informs that clinical trials and case reports did not establish any risk association between remdesivir and pregnancy. Studies in animals showed that the medicine did not cause adverse events related to the foetus. The document also states that guidance regarding lactation is related to the COVID-19 exposure protocols. There is no data on presence of the medicine in human breast milk, although it is in fact found in animals (Veklury, 2023).

The review by Jorgensen, Davis, and Lapinsky (2021) presents some findings that link remdesivir use in pregnant women to miscarriages and preterm deliveries. However, the available data were insufficient to establish the potential adverse events related to remdesivir.

COVID-19 is associated with an increased risk of preterm delivery and neonatal morbidity (Allotey et al., 2020), which may explain some of the signals identified. Adverse pregnancy and neonatal outcomes are more common in pregnant individuals infected with coronavirus, particularly if the disease presents itself in a severe form (Jamieson and Rasmussen, 2022). The gestational trimester in which a pregnant woman is infected also impacts the pregnancy outcome. Data indicate that infection during the last trimester of pregnancy is significantly associated with preterm births (Fallach et al., 2022).

On the other hand, some data indicate that pregnancy is also a risk factor for COVID-19 (Zambrano et al., 2020), although more recent studies suggest that the current data are insufficient to conclude this correlation. Furthermore, many studies indicate that pregnant women experience more severe cases of the disease than non-pregnant women (Jamieson and Rasmussen, 2022).

Other factors such as age, underlying conditions (obesity, hypertension, chronic lung diseases) and gestational diabetes are associated with more severe COVID-19 cases during pregnancy (Galang et al., 2021).

However, the findings (Figure 2) correlate the age range from 30 to 39 years old more strongly to significant adverse events related to pregnancy. This might be a reflection of a higher number of pregnant individuals belonging to this age group or an indication that the events are indeed related to the medicine and not to COVID-19 severity.

The study by Tavakoli et al. (2023) showed the benefit of using remdesivir to reduce vertical transmission of the disease. This observation may explain why “maternal exposure during pregnancy” had the highest frequency among all pregnancy-related signals for remdesivir (*n* = 70).

### 4.3 Comparison of signals to product labels

None of the possible signals related to pregnancy and the foetus generated for remdesivir and tocilizumab are mentioned in the product labels.

### 4.4 Database characteristics and limitations

It is known that databases of spontaneous adverse drug event reporting systems suffer from underreporting; a tendency to notify more on severe or rare events than others; and the reporting of events related to newly marketed medicines (Alomar et al., 2020), which can affect data mining.

Duplication of reports in spontaneous reporting databases is a common issue that can interfere with data analysis. In this study, we performed a database debugging process solely with respect to the report identification key (Case ID field), disregarding other fields that might remove more duplicates, as indicated by Poluzzi et al. (2012).

For both medicines analyzed, use indications that are not described in their respective labels were linked, such as hypertension and thrombotic prophylaxis (for remdesivir) and diabetes and anxiety (for tocilizumab). Although these uncommon associations represent a small percentage of the use indications, this finding may indicate certain lack of clarity among the notifiers regarding how to accurately fill out report fields.

Due to the data amount, prior harmonization of the database at the “Preferred Term” level, and the exploratory nature of the study, it was decided not to combine terms before calculating ROR. Subsequent clinical analysis may be necessary to determine whether the potential signals found are redundancies or clinically distinct.

Many signals found in the database do not represent potential adverse reactions but, rather, medical events related to the patient or clinical procedures, such as “clinical trial participant” and “tracheal aspirate culture”, respectively.

The MedDRA terminology applies to various medicine development stages, addressing everything from adverse health effects to device malfunctions. In addition to signs and symptoms, other categories of medical terms include diagnoses, medication errors, medical procedures and medical/social/family history. Even if they are not adverse reactions, this range of categories is applied to a report when relevant for regulatory data assessment (MedDRA, 2020).

As is the case with other pharmacovigilance databases, it is not always possible to obtain certain clinical data about the patients, such as gestational age at the time of the reported adverse event and presence of underlying conditions that are not related to the indicated use of the medicine reported as primary suspect.

Nevertheless, signals reflecting use of these drugs during pregnancy are important findings to raise awareness about potential adverse reactions for this population. As pregnant individuals are typically excluded from clinical trials, the teratogenic effects rely on pharmacovigilance studies for identification (Cavadino et al., 2021).

The higher proportion of missing data for biological sex and age at the time of the adverse events for tocilizumab when compared to remdesivir might indeed be related to the fact that many notifiers are consumers (Table 2), unlike the latter, which is typically administered in a healthcare setting (Table 1). Consumer-reported data may be more likely to have missing or incomplete information, which might explain the difference in data completeness between both medicines.

Consumers have proved to be active agents in reporting ADEs to FAERS. The FDA has a history of encouraging medicine users to participate in clinical trials and drug approval processes (Kieffer et al., 2019; Kruger and Gasparini, 2020). The systematic review by Inácio et al. (2016) identified that patient reports might be essential for discovering ADEs in specific populations that do not participate in clinical trials.

Tocilizumab is an example of the importance of patient reporting. One possible reason is its use in treating rheumatic diseases, as chronic patients tend to play a more active role in pharmacovigilance (Kruger and Gasperini, 2020). Another important factor is the pharmaceutical form of the medicines: tocilizumab can be administered subcutaneously by the patients themselves, whereas remdesivir is exclusively administered intravenously, requiring a health professional for the procedure.

Lastly, the signals related to the medicines found in this study reflect a correlation. Clinical studies are required to establish causality between potential adverse reactions and medicine use (Bihan et al., 2020).

The current study focused on data from both years of the COVID-19 pandemic. However, it would be interesting for future research to explore the association of tocilizumab with events related to pregnancy and the foetus during the period before the COVID-19 pandemic in the FAERS database, where the only indicated use in the label would be for rheumatological diseases. The degree of data incompleteness and inconclusive data regarding use indications becomes a potential confounding bias.

The automation level can also be improved, especially if the goal is to compare signals from different periods. It is possible to perform signal detection entirely through the Grafana platform, although data related to the System Organ Class (SOC) needs to be collected manually because it is not included as a report field in the quarterly dataset publicly provided by FAERS.

## 5 Conclusion

Remdesivir and tocilizumab are used in the in-hospital treatment for COVID-19, even for pregnant individuals. Our study identified 303 possible safety signals for remdesivir and 578 for tocilizumab, of which 6 and 3 (respectively) were related to pregnancy, foetuses and neonates. These adverse events were not found in the label’s product.

The underlying conditions listed in the indications for both medicines are related to adverse pregnancy and infant outcomes, becoming a confounding factor in the signal analysis. Nonetheless, the associations generated in this study shed light on medicine use safety during pregnancy and can guide clinical studies to determine causality of these events.

## Data Availability

The original contributions presented in the study are included in the article/Supplementary Material, further inquiries can be directed to the corresponding authors.
