# Supplementary material for: Detection of potential safety signals related to the use of remdesivir and tocilizumab in the COVID era during pregnancy, resorting to open data from the FDA adverse event reporting system (FAERS)
Source: Front Pharmacol. 2024 Jan 31;15:1349543. doi: 10.3389/fphar.2024.1349543 (PMC10870234; doi:10.3389/fphar.2024.1349543)
Supplement: Supplementary file 1 [file DataSheet1.docx]

Supplementary Material

# Supplementary Data

GitHub link related to the creation of tables with FAERS data (2020-2022):
<https://github.com/jokerfvd/FAERS2DB/blob/main/create-tables.sql>

GitHub link for the Ruby program created to read TXT files and insert data into the database: <https://github.com/jokerfvd/FAERS2DB/blob/main/FAERS2DB.rb>

Snapshots of the dashboards created in Grafana for the analysis of Remdesivir-related notification data:

ROR Calculation:
https://snapshots.raintank.io/dashboard/snapshot/CA0HXYrBplcDA0Bk1UiFvoTzvrt7RnYN?orgId=2

Other Report Data:
https://snapshots.raintank.io/dashboard/snapshot/RMZcVdV1uzCrF4wpF9MPLT7fX4Od1xmg?orgId=2

Analysis of the notifications that originated the signals:
https://snapshots.raintank.io/dashboard/snapshot/IAcndcL578xky1uQAtbDPTJPbCkf5a3B?orgId=2

Snapshots of the dashboards created in Grafana for the analysis of Tocilizumab-related notification data:

ROR Calculation:
https://snapshots.raintank.io/dashboard/snapshot/QWwG9ajc2LDdbBv8hg0QhJkgmBcHIiFz?orgId=2

Other Report Data:
https://snapshots.raintank.io/dashboard/snapshot/b4S0PaMRaXsXcTvo0P6do7R7YYRaMkcV?orgId=2

Analysis of the notifications that originated the signals:
https://snapshots.raintank.io/dashboard/snapshot/3AbiKnclhuXQI2VRoDBGUbRc5yx7BCLP?orgId=2
